# Infratubercle Anterior Closing Wedge Osteotomy Corrects Sagittal Alignment without Affecting Coronal Alignment or Patellar Height

**DOI:** 10.3390/jcm13164715

**Published:** 2024-08-11

**Authors:** Shintaro Onishi, Youngji Kim, Hiroshi Nakayama, Alfred A. Mansour, Walter R. Lowe, Matthieu Ollivier

**Affiliations:** 1 Institute for Locomotion, Aix-Marseille University, Assistance Publiqu-Hôpitaux de Marseille, Centre National de la Recherche Scientifique, Institut des Sciences du Mouvement, Sainte-Marguerite Hospital, 13009 Marseille, France; 0024shintaro@gmail.com (S.O.); yonji@juntendo.ac.jp (Y.K.); 2 Department of Orthopedic Surgery, Hyogo Medical University, Nishinomiya 6638501, Japan; hiroshi0273@mac.com; 3 Department of Orthopaedics, Juntendo University, Tokyo 1130034, Japan; 4 Department of Orthopedic Surgery, University of Texas Health Science Center at Houston, Houston, TX 77401, USA; alfred.a.mansour@uth.tmc.edu (A.A.M.III); walter.r.lowe@uth.tmc.edu (W.R.L.)

**Keywords:** infratubercle, anterior closing wedge osteotomy, slope reducing osteotomy, anterior cruciate ligament, posterior tibial slope, patellar height

## Abstract

**Background:** Excessive posterior tibial slope (PTS) has been associated with a higher risk of graft failure after anterior cruciate ligament reconstruction (ACLR). Although anterior closing wedge osteotomy (ACWO) can reduce the PTS, it may also change the coronal alignment and patellar height. **Purpose:** To elucidate the radiological outcomes after infratubercle ACWO, specifically to evaluate its influence on perioperative changes in patellar height. **Methods:** Patients who underwent infratubercle ACWO with combined ACLR with a minimum follow-up of 3 months were included. Surgery was indicated when the PTS was greater than 12°. Radiological evaluation included measurements of the hip–knee–ankle angle (HKA), PTS, femoral patellar height index (FPHI), and Caton–Deschamps index (CDI) preoperatively and 3 months postoperatively. Patellar height was classified as patella baja, normal, or alta based on CDI values. Knee recurvatum was measured preoperatively and at final follow-up. **Results:** A total of 21 patients with a mean age of 21.6 ± 3.0 years were included. Although HKA did not significantly change, significant corrections were achieved in the PTS from 14.5° ± 1.6° to 5.7° ± 1.0° (*p* < 0.001). No significant change in FPHI was found (preoperative: 1.33 ± 0.11 vs postoperative: 1.30 ± 0.09). Patellar height categories showed no significant differences pre- and postoperatively, while three patients (14.3%) changed their patellar height category (all moved up one category). Knee recurvatum increased significantly from 4.9° ± 2.9° preoperatively to 7.8° ± 3.1° at the final follow-up (*p* < 0.001). **Conclusions:** Precise sagittal correction was achieved after infratubercle ACWO without altering the coronal alignment and patella height. Level of Evidence: IV, Case series.

## 1. Introduction

Anterior cruciate ligament (ACL) injuries are common in the young athletic population due to intrinsic or extrinsic factors. Numerous studies have investigated clinical outcomes after anatomic ACL reconstruction (ACLR); however, a higher incidence of graft failure or graft rupture has still been reported [1,2,3,4,5,6,7,8]. Intrinsic risk factors for ACL injuries include age, gender, genetic, family history, and anatomical pathologies such as bone morphology and joint laxity [1,5,6,7,8]. Among those factors, the posterior tibial slope (PTS) is known to be an anatomical risk factor for the failure of ACLR [5,9,10]. To date, PTS values greater than 12° have been associated with a higher risk of graft rupture after ACLR [9,11]. Previous biomechanical studies have demonstrated the influence of an excessive PTS on anterior tibial translation (ATT) under axial load [12], and slope-reducing osteotomy can reduce either ACL or ACL-graft force [13,14]. Consequently, slope-reducing anterior closing wedge osteotomy (ACWO) has been employed for ACL-injured patients with a higher PTS to reduce the excessive PTS and prevent further graft failure: graft failure is a great matter of interest, and research is trying to reduce the incidence with biomaterials [15,16,17,18].

ACWO corrects sagittal alignment, but recent studies indicate it can significantly decrease the medial proximal tibial angle (MPTA) in the coronal plane, potentially causing postoperative tibial varus [19,20]. Reducing MPTA should be avoided despite ACWO’s sagittal correction capability.

Postoperative anterior knee pain, instability, and stiffness related to patellar height changes can lead to patellofemoral osteoarthritis [21,22,23,24,25,26]. ACWO may change patellar height due to sagittal plane correction. Various ACWO techniques, including supratubercle, transtuberosity, and infratubercle, have been studied for their effects on patellar height [17,27,28,29,30,31,32,33,34]. However, most research has focused on supratubercle ACWO, and little information is available about infratubercle ACWO’s impact on patellar height.

This study aims to elucidate radiological outcomes after infratubercle ACWO, focusing on its influence on coronal and sagittal alignment and patellar height.

## 2. Methods

### 2.1. Study Population and Design

**Figure 1:**
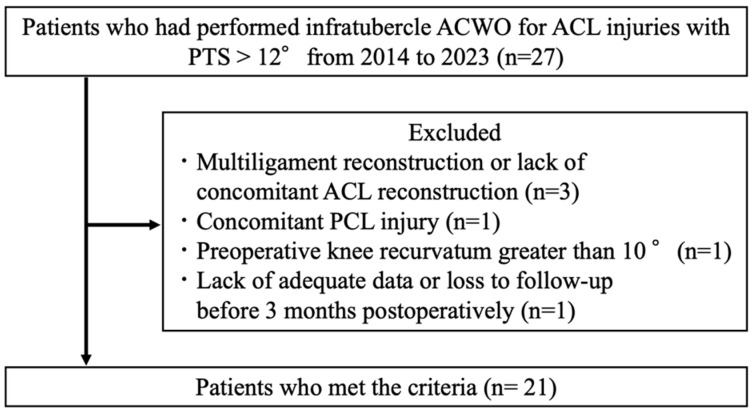
Flowchart of the patient selection process. ACWO, anterior closing wedge osteotomy; ACL, anterior cruciate ligament; PCL, posterior cruciate ligament; PTS, posterior tibial slope.

After institutional review board approval (approval code: PADS24-177_dgr), a retrospective analysis of a prospectively maintained database was performed on a consecutive series of infratubercle ACWO with combined ACLR between 2014 and 2023. Inclusion criteria included concomitant ACLR with infratubercle ACWO and pre- and 3 months postoperative radiographs. Exclusion criteria included multi-ligament reconstruction or lack of concomitant ACLR, concomitant posterior cruciate ligament injury, significant preoperative knee recurvatum greater than 10° and inadequate data or loss to follow-up before 3 months postoperatively. The patient flowchart is presented in Figure 1.

### 2.2. Surgical Options and Procedures

Surgeries were performed by a single senior surgeon at a tertiary referral center. Surgery was indicated when the PTS was greater than 12° as measured by preoperative whole leg lateral radiographs. Radiological evaluation and surgical planning were conducted using both whole leg and standard knee radiographs. The intended PTS was set at 4° to 6°. Intended correction angle and gap distance were calculated from preoperative radiographs in all cases.

**Figure 2:**
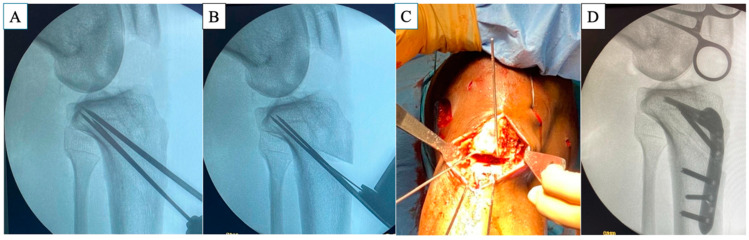
Sequential steps of infratubercle ACWO in a representative case. (**A**) K-wires are inserted from distal to the tibial tubercle toward the posterior cortex according to the tibial insertion of the posterior cruciate ligament under adequate fluoroscopic control. (**B**,**C**) After osteotomy, the anterior bone wedge is removed. (**D**) Plate fixation is then performed to maintain the corrected PTS. ACWO, anterior closing wedge osteotomy; PTS, posterior tibial slope.

Surgeries were performed under general or spinal anesthesia with a pneumatic tourniquet. First, if an autograft was chosen, the graft was harvested and prepared for ACLR. Arthroscopic examination and intra-articular pathology procedures, such as meniscal and chondral lesions, were performed as needed. The femoral bone tunnel was drilled via the inside–out technique with diameters corresponding to the graft size. The infratubercle ACWO procedure was then performed. An anterior approach to the proximal tibia with a longitudinal skin incision and exposure for ACWO was performed. The superficial medial collateral ligament was minimally released for medial exposure, and the tibialis anterior muscle was released for lateral exposure. Two K-wires were inserted from just distal to the tibial tubercle toward the posterior cortex under fluoroscopic control. Additional K-wires were inserted distally according to preoperatively measured distances. The osteotomy was performed using an oscillating saw (Precision^®^, Stryker, Bruz, France), avoiding breaking the posterior hinge. The anterior bone wedge was removed, and the osteotomy site was closed by compressing the proximal tibia. The intended PTS was achieved under fluoroscopic control, and the Activmotion^®^ HTO plate (Newclip Technics, Haute Goulaine, France) was used to maintain the osteotomy site (Figure 2). After the osteotomy, the tibial bone tunnel for ACLR was created. Finally, ACLR using either autograft or bone–patellar tendon–bone allograft was performed with lateral extra-articular tenodesis using modified Lemaire techniques [35]. The graft choice was based on the number of ACLR and the previous graft source.

### 2.3. Postoperative Rehabilitation

Quadriceps activation including isolated quadriceps contraction, cryotherapy, and electric muscle stimulation was initiated immediately to prevent arthrogenic muscle inhibition [36,37]. The range of motion was allowed to 90 degrees of flexion for 3 weeks and then progressed to no restriction. A brace was recommended for approximately 6 weeks with a −10° extension restriction. Weight bearing was restricted for 3 weeks. Partial weight bearing with crutches then started at 3 weeks, progressed to full weight bearing based on radiographic healing of the osteotomy site, typically at 6 weeks. Closed kinetic chain muscle strength exercises were mainly applied for 3 months, while open kinetic chain muscle strengthening exercises were contraindicated to reduce the risk of tubercle fracture and dislocation of the anterior osteotomy site until 3 months postoperatively. Jogging and recreational non-pivoting sports were allowed at 4 months, and return to pivoting sports was recommended after adequate muscle strength and solid bony healing at the osteotomy site, which was typically at least 8 months postoperatively.

### 2.4. Radiographic Measurements and Clinical Outcomes

**Figure 3:**
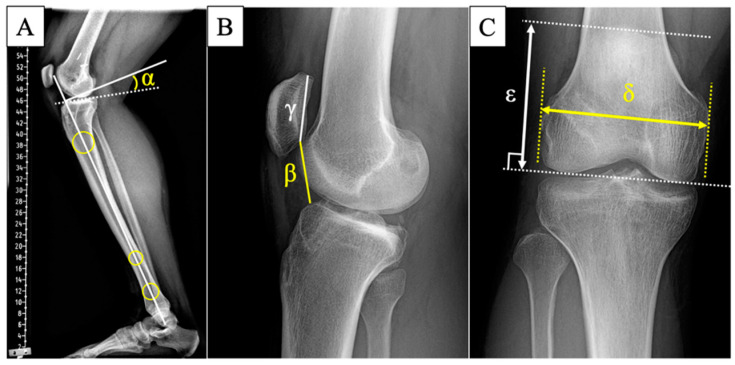
Measurement of radiographic parameters using plain radiograph. (**A**) posterior tibial slope = α. (**B**) Caton–Deschamps index = β/γ. (**C**) femoral patellar height index = δ/ε.

Pre- and postoperative radiological and clinical data were retrospectively reviewed and analyzed. Radiological evaluations included measurements of the hip–knee–ankle angle (HKA), posterior tibial slope (PTS), femoral patellar height index (FPHI), Caton–Deschamps index (CDI), and knee recurvatum preoperatively and 3 months postoperatively. The HKA and PTS were assessed using whole leg radiographs, while CDI and FPHI were measured on conventional knee lateral radiographs (Figure 3). Coronal whole leg radiographs were assessed for HKA. Lateral long-leg radiographs, considered more accurate for assessing the PTS than short radiographs, were used [38,39]. The PTS was defined as the angle between the tangent line of the medial tibial plateau and the line perpendicular to the tibial axis, which was indicated by three circles that best fit within the anterior and posterior tibial cortices over the full length of the tibia [40]. Patellar height was measured using the femoral-referenced method of FPHI proposed by Ihle and Schröter et al. [41] using AP knee radiographs. Radiographs were taken at 5° of knee flexion and considered adequate if the cortical margins of the posterior aspect of the medial and lateral femoral condyles were superimposed. Finally, the height of the patella was subdivided into three categories (alta, baja, and normal) based on the tibial-referenced method of CDI [42].

Patellar height was classified as patella baja, normal, or alta according to CDI values both pre- and postoperatively. A CDI of 0.8 to 1.2 indicates normal patellar height, while a CDI > 1.2 indicates patella alta, and a CDI < 0.8 indicates patella baja [42,43]. Although FPHI has been validated with good to excellent intra- and inter-rater reliability [28,41,44], there are no globally accepted criteria for FPHI, so it was not used to categorize patellar height. Knee recurvatum was measured with a goniometer preoperatively and at a final follow-up.

### 2.5. Statistical Analysis

All statistical analyses were performed using SPSS version 18 (SPSS Inc., Chicago, IL, USA). The normality of data distribution was assessed using the Shapiro–Wilk test. Statistical comparisons between pre- and postoperative values were made using the paired *t*-test for parametric data, the Wilcoxon signed-rank test for non-parametric data, and the Chi-squared test for categorical outcomes with significance set at or below 0.05. Measurement accuracy was assessed using the intraclass correlation coefficient for intra- and interobserver reliability. Two independent observers reviewed preoperative and postoperative radiographs twice in a blinded fashion with a 3-week measurement interval; all intraclass and interclass correlation coefficients were >0.8. To assess the statistical power of this study, a post hoc power analysis was conducted for comparison of the pre- and postoperative PTS using a two-sided test. Consequently, it was shown that the required total sample size of 21 in this study could achieve an adequate power 1 − β of 0.991 with an α of 0.05 and effect size of 6.29, using G-Power (version 3.1.9.2; Franz Faul, Universität Kiel).

## 3. Results

**Figure 4:**
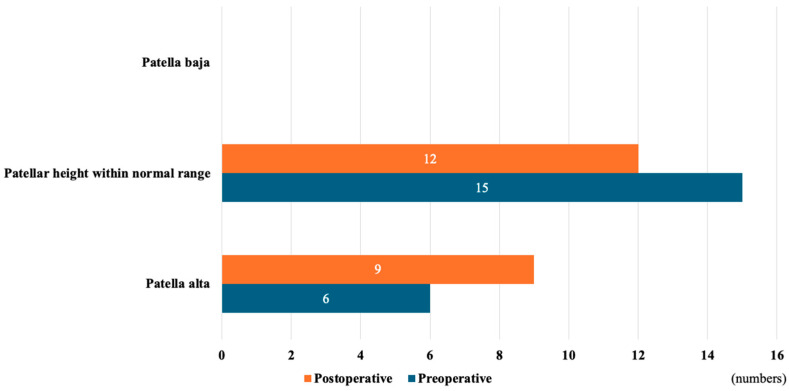
Patellar height classification according to the Caton–Deschamps index.

A total of 21 patients (n = 21 knees) with a mean age of 21.6 ± 3.0 years were included. Patient demographics are shown in Table 1. Significant pre- to postoperative corrections were achieved in the PTS from 14.5° ± 1.6° to 5.7° ± 1.0° (*p* < 0.001). Meanwhile, HKA did not significantly change during the study period (preoperative: 178.3° ± 2.6° vs. postoperative: 179.2° ± 1.1°, *p* = 0.147). Regarding patella height, no significant change in FPHI was found (preoperative: 1.33 ± 0.11 vs postoperative: 1.30 ± 0.09, *p* = 0.103) (Table 2). Patellar height categories showed no significant differences pre- and postoperatively (*p* = 0.627), while three patients (14.3%) changed their patellar height category according to the CDI value (all moved up one category) (Figure 4).

Knee recurvatum significantly increased from 4.9° ± 2.9° preoperatively to 7.8° ± 3.1° at the final follow-up (*p* < 0.001) (Table 3).

## 4. Discussion

The main finding of this study is that infratubercle ACWO can correct precise sagittal alignment without altering coronal alignment in patients with recurrent ACL injuries and a high PTS. No significant changes were observed using the femoral reference method of FPHI (preoperative: 1.33 ± 0.11 vs. postoperative: 1.30 ± 0.09, *p* = 0.103). Moreover, there were no significant changes in patellar height categories before and after infratubercle ACWO.

An excessive PTS can lead to excessive ATT and increased posterior tibiofemoral contact pressures [12,13,45]. Studies have shown that an increased PTS is associated with a higher risk of further ACL injuries after ACLR [5,9]. Previous studies have also demonstrated that ACWO allows significant reductions in ATT and ACL forces [12,13,14]. In line with previous biomechanical studies, satisfactory clinical outcomes of ACWO procedures have been reported even in patients with multiple ACLR failures [15,16,18,46].

A recent study demonstrated that ACWO can decrease the medial proximal tibial angle (MPTA) in the coronal plane, resulting in unintentional postoperative tibial varus [19,20]. Previous cohort studies have shown that patients with multiple ACLR failures are more likely to suffer from chondral injuries in the medial compartment [47]. Therefore, despite ACWO’s goal of sagittal alignment correction, unintentional varus alignment should be avoided. This study showed that accurate sagittal alignment corrections were achieved without significantly altering the coronal alignment. However, the mean HKA increased by 0.9° in this cohort. Although this value is small, the clinical significance of this difference should be evaluated in further studies. 

Changes in patellar height are a major concern after osteotomies around the knee. Numerous studies have investigated changes in patellar height, particularly after high tibial osteotomy (HTO) [48,49,50,51]. Generally, the patellar height tends to decrease after the medial opening wedge HTO, whereas it remains unchanged after the lateral closing wedge HTO. However, there is limited evidence regarding changes in patellar height after ACWO. Cance et al. reported that CDI increased by a mean of +0.08 after supratuberosity ACWO [52]. Conversely, Guy et al. found no significant difference in patellar height indices, such as the Insall–Salvati index, CDI, and FPHI, before and after supratubercle ACWO. Changes in patellar height categories (baja, normal, alta) occurred in both directions in their study. Despite conflicting findings, several studies have shown a proportional correlation between a delta PTS and either delta CDI or CDI ratio (preoperative value/postoperative value) [28,32].

**Figure 5:**
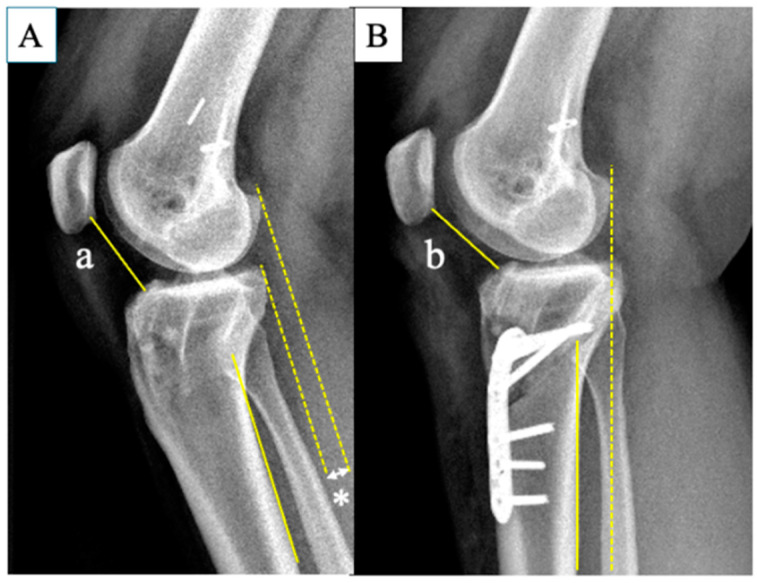
A representative case of a patient who underwent combined infratubercle ACWO with revision ACL reconstruction. (**A**) Preoperative plain lateral knee radiograph showing anterior tibial translation relative to the femur (*) due to ACL deficiency and an excessively high PTS. (**B**) Anterior tibial translation is reduced after surgery. Consequently, distance “a” increases slightly to distance “b” due to the reduction in anterior tibial translation after surgery. ACWO, anterior closing wedge osteotomy; ACL, anterior cruciate ligament; PTS, posterior tibial slope.

To date, there are no relevant studies on changes in patellar height before and after infratubercle ACWO. Theoretically, infratubercle ACWO should not change the patellar height because the patellar tendon insertion and tibial tuberosity are preserved. Patellar height categories showed no significant differences pre- and postoperatively in the present study, while three patients (14.3%) moved up their patellar height category according to the CDI value. A possible explanation is the reduction in anterior tibial translation (ATT) after ACWO. Luceri et al. reported that abnormal ATT in ACL-deficient knees lowers patellar height [53]. They demonstrated that the distance between the inferior patellar pole and the upper tibia shortens with abnormal ATT. In patients with a high PTS requiring ACWO, ATT relative to the femur is common due to ACL insufficiency and an excessively high PTS. ACWO reduces ATT by correcting the PTS [17,34], increasing the distance between the inferior patella and anterior tibia (Figure 5). Since posterior tibial reduction after ACWO does not affect femoral parameters, the lack of significant change in the FPHI method in this study is reasonable. This raises concerns about the validity of CDI for evaluating patellar height in this population. Based on this study, the effect of infratubercle ACWO on patellar height is limited in clinical settings.

Iatrogenic postoperative knee recurvatum is another concern after slope-reducing osteotomy. Excessive knee recurvatum can lead to knee pain, impairment of the extensor mechanism, extension gait patterns, and stretching of posterior knee structures [54,55,56]. High degrees of knee recurvatum are associated with poor clinical outcomes after ACLR [57,58]. Clinical studies have shown that ACWO can increase knee recurvatum in exchange for the PTS reduction [16,30,46]. In this study, the mean PTS reduction after infratubercle ACWO was approximately 8.8°, while the mean knee recurvatum increased by 2.9°. Similarly, Mabrouk et al. reported a mean change in knee recurvatum of 2.6° after supratubercle ACWO despite a 9.5° PTS correction [16]. Rozinthe et al. conducted a long-term follow-up study (mean follow-up of 9.9 years) and found that one in nine patients experienced 10° of knee recurvatum [46]. Most patients in previous studies were asymptomatic, but care must be taken to avoid excessive postoperative knee recurvatum.

## 5. Limitations

Several limitations exist in this study. It is a retrospective case series with short-term follow-up and a small sample size. Patellar height indices were measured on lateral radiographs taken at 5° of flexion, not at 30° as originally described. However, all patients were assessed using the same protocol during the study period. The study focused on patellar height without assessing other patellofemoral joint parameters or correlating radiographic findings with clinical outcomes. Therefore, the clinical significance of changes in patellar height remains unclear. Additionally, there was no control group. Despite these limitations, this is the first study to investigate the influence of infratubercle ACWO on patellar height in ACL-injured patients with a high PTS. Further comparative studies with longer follow-ups and larger sample sizes are needed to elucidate the influence of various ACWO procedures on the patellofemoral joint.

## 6. Conclusions

Precise sagittal correction was achieved after infratubercle ACWO without altering the coronal alignment and patella height. No significant changes were observed using the femoral reference method of FPHI. Moreover, there were no significant changes in patellar height categories before and after infratubercle ACWO. Further comparative studies are needed to elucidate the influence of various ACWO procedures on the patellofemoral joint.

## Figures and Tables

**Table 1:** Clinical characteristics. Values are expressed as mean and standard deviations with ranges in parentheses. BMI, body mass index; ACL, anterior cruciate ligament.

	Overall (n = 21)
Age (years)	21.6 ± 3.0 (16–31)
Male/Female (Female, %)	7/14 (66.7%)
BMI (kg/m^2^)	22.8 ± 2.5 (19–28)
Side (Right/Left)	11/10
Follow-up period (month)	9.9 ± 3.0 (3–14)
Number of ACL reconstruction (1/2/3, %)	1/13/7 (4.8/61.9/33.3)
Autograft/Allograft (autograft, %)	16/5 (76.2%)

**Table 2:** Comparison between pre- and postoperative radiological parameters. Values are expressed as mean and standard deviations.

		Overall (n = 21)
HKA (degree)	Preoperative	178.3 ± 2.6
	Postoperative	179.2 ± 1.1
*p* value *		0.147
PTS (degree)	Preoperative	14.5 ± 1.6
	Postoperative	5.7 ± 1.0
*p* value *		<0.001
FPHI	Preoperative	1.33 ± 0.11
	Postoperative	1.30 ± 0.09
*p* value *		0.103

* Statistical comparison between the pre- and postoperative parameters. HKA, hip–knee–ankle angle; PTS, posterior tibial slope; FPHI, femoral patella height index.

**Table 3:** Pre- and postoperative degree of knee recurvatum. Values are expressed as mean and standard deviations. * Statistical comparison between the pre- and postoperative parameters.

		Overall (n = 21)
Knee recurvatum (degree)	Preoperative	4.9 ± 2.9
	Postoperative	7.8 ± 3.1
*p* value *		<0.001
Knee recurvatum (degree)	Contralateral side	3.6 ± 2.9

## Data Availability

Data is unavailable due to privacy or ethical restrictions.

## References

[1] Diquattro E., Jahnke S., Traina F., Perdisa F., Becker R., Kopf S. (2023). ACL surgery: Reasons for failure and management. EFORT Open Rev..

[2] Webster K.E., Feller J.A. (2016). Exploring the High Reinjury Rate in Younger Patients Undergoing Anterior Cruciate Ligament Reconstruction. Am. J. Sports Med..

[3] Della Villa F., Hagglund M., Della Villa S., Ekstrand J., Walden M. (2021). High rate of second ACL injury following ACL reconstruction in male professional footballers: An updated longitudinal analysis from 118 players in the UEFA Elite Club Injury Study. Br. J. Sports Med..

[4] Barber-Westin S., Noyes F.R. (2020). One in 5 Athletes Sustain Reinjury Upon Return to High-Risk Sports After ACL Reconstruction: A Systematic Review in 1239 Athletes Younger Than 20 Years. Sports Health.

[5] Salmon L.J., Heath E., Akrawi H., Roe J.P., Linklater J., Pinczewski L.A. (2018). 20-Year Outcomes of Anterior Cruciate Ligament Reconstruction with Hamstring Tendon Autograft: The Catastrophic Effect of Age and Posterior Tibial Slope. Am. J. Sports Med..

[6] Sanders T.L., Pareek A., Hewett T.E., Levy B.A., Dahm D.L., Stuart M.J., Krych A.J. (2017). Long-term rate of graft failure after ACL reconstruction: A geographic population cohort analysis. Knee Surg Sports Traumatol. Arthrosc..

[7] Maletis G.B., Inacio M.C., Funahashi T.T. (2015). Risk factors associated with revision and contralateral anterior cruciate ligament reconstructions in the Kaiser Permanente ACLR registry. Am. J. Sports Med..

[8] Cruz A.I., Beck J.J., Ellington M.D., Mayer S.W., Pennock A.T., Stinson Z.S., VandenBerg C.D., Barrow B., Gao B., Ellis H.B. (2020). Failure Rates of Autograft and Allograft ACL Reconstruction in Patients 19 Years of Age and Younger: A Systematic Review and Meta-Analysis. JBJS Open Access.

[9] Webb J.M., Salmon L.J., Leclerc E., Pinczewski L.A., Roe J.P. (2013). Posterior tibial slope and further anterior cruciate ligament injuries in the anterior cruciate ligament-reconstructed patient. Am. J. Sports Med..

[10] Shelbourne K.D., Benner R.W., Jones J.A., Gray T. (2021). Posterior Tibial Slope in Patients Undergoing Anterior Cruciate Ligament Reconstruction with Patellar Tendon Autograft: Analysis of Subsequent ACL Graft Tear or Contralateral ACL Tear. Am. J. Sports Med..

[11] Lee C.C., Youm Y.S., Cho S.D., Jung S.H., Bae M.H., Park S.J., Kim H.W. (2018). Does Posterior Tibial Slope Affect Graft Rupture following Anterior Cruciate Ligament Reconstruction?. Arthroscopy.

[12] Giffin J.R., Vogrin T.M., Zantop T., Woo S.L., Harner C.D. (2004). Effects of increasing tibial slope on the biomechanics of the knee. Am. J. Sports Med..

[13] Imhoff F.B., Mehl J., Comer B.J., Obopilwe E., Cote M.P., Feucht M.J., Wylie J.D., Imhoff A.B., Arciero R.A., Beitzel K. (2019). Slope-reducing tibial osteotomy decreases ACL-graft forces and anterior tibial translation under axial load. Knee Surg. Sports Traumatol. Arthrosc..

[14] Yamaguchi K.T., Cheung E.C., Markolf K.L., Boguszewski D.V., Mathew J., Lama C.J., McAllister D.R., Petrigliano F.A. (2018). Effects of Anterior Closing Wedge Tibial Osteotomy on Anterior Cruciate Ligament Force and Knee Kinematics. Am. J. Sports Med..

[15] Dejour D., Saffarini M., Demey G., Baverel L. (2015). Tibial slope correction combined with second revision ACL produces good knee stability and prevents graft rupture. Knee Surg. Sports Traumatol. Arthrosc..

[16] Mabrouk A., Kley K., Jacquet C., Fayard J.M., An J.S., Ollivier M. (2023). Outcomes of Slope-Reducing Proximal Tibial Osteotomy Combined with a Third Anterior Cruciate Ligament Reconstruction Procedure with a Focus on Return to Impact Sports. Am. J. Sports Med..

[17] Song G.Y., Ni Q.K., Zheng T., Zhang Z.J., Feng H., Zhang H. (2020). Slope-Reducing Tibial Osteotomy Combined with Primary Anterior Cruciate Ligament Reconstruction Produces Improved Knee Stability in Patients with Steep Posterior Tibial Slope, Excessive Anterior Tibial Subluxation in Extension, and Chronic Meniscal Posterior Horn Tears. Am. J. Sports Med..

[18] Baldwin P., Li D.J., Auston D.A., Mir H.S., Yoon R.S., Koval K.J. (2019). Autograft, Allograft, and Bone Graft Substitutes: Clinical Evidence and Indications for Use in the Setting of Orthopaedic Trauma Surgery. J. Orthop. Trauma.

[19] Mayer P., Schuster P., Schlumberger M., Michalski S., Gesslein M., Beel W., Immendorfer M., Richter J. (2023). Effect of Anterior Tibial Closing Wedge Osteotomy on Coronal Tibial Alignment in Relation to Preoperative Medial Proximal Tibial Angle and Wedge Height. Am. J. Sports Med..

[20] Weiler A., Gwinner C., Wagner M., Ferner F., Strobel M.J., Dickschas J. (2022). Significant slope reduction in ACL deficiency can be achieved both by anterior closing-wedge and medial open-wedge high tibial osteotomies: Early experiences in 76 cases. Knee Surg. Sports Traumatol. Arthrosc..

[21] Luyckx T., Didden K., Vandenneucker H., Labey L., Innocenti B., Bellemans J. (2009). Is there a biomechanical explanation for anterior knee pain in patients with patella alta?: Influence of patellar height on patellofemoral contact force, contact area and contact pressure. J. Bone Jt. Surg. Br. Vol..

[22] Ambra L.F., Hinckel B.B., Arendt E.A., Farr J., Gomoll A.H. (2019). Anatomic Risk Factors for Focal Cartilage Lesions in the Patella and Trochlea: A Case-Control Study. Am. J. Sports Med..

[23] Fitzpatrick C.K., Steensen R.N., Tumuluri A., Trinh T., Bentley J., Rullkoetter P.J. (2016). Computational analysis of factors contributing to patellar dislocation. J. Orthop. Res..

[24] Vives-Barquiel M.A., Torrents A., Lozano L., Martinez-Pastor J.C., Macule F., Segur J.M., Popescu D. (2015). Proximalize osteotomy of tibial tuberosity (POTT) as a treatment for stiffness secondary to patella baja in total knee arthroplasty (TKA). Arch. Orthop. Trauma Surg..

[25] Stefanik J.J., Guermazi A., Zhu Y., Zumwalt A.C., Gross K.D., Clancy M., Lynch J.A., Segal N.A., Lewis C.E., Roemer F.W. (2011). Quadriceps weakness, patella alta, and structural features of patellofemoral osteoarthritis. Arthritis Care Res..

[26] Haj-Mirzaian A., Guermazi A., Pishgar F., Pourvaziri A., Roemer F.W., Sereni C., Hakky M., Zikria B., Stefanik J.J., Demehri S. (2019). Association of patella alta with worsening of patellofemoral osteoarthritis-related structural damage: Data from the Osteoarthritis Initiative. Osteoarthr. Cartil..

[27] Dan M.J., Cance N., Pineda T., Demey G., Dejour D.H. (2024). Four to 6° Is the Target Posterior Tibial Slope After Tibial Deflection Osteotomy According to the Knee Static Anterior Tibial Translation. Arthroscopy.

[28] Guy S., Saithna A., Ferreira A., Carrozzo A., Vieira T.D., Ollivier M.P., Sonnery-Cottet B. (2024). The Influence of Tibial Tubercle-Sparing Slope-Reducing Osteotomy on Patellar Height in Patients Undergoing Revision ACL Reconstruction. Am. J. Sports Med..

[29] Floyd E.R., Carlson G.B., Monson J., LaPrade R.F. (2021). Tibial Tubercle Preserving Anterior Closing Wedge Proximal Tibial Osteotomy and ACL Tunnel Bone Grafting for Increased Posterior Tibial Slope in Failed ACL Reconstructions. Arthrosc. Tech..

[30] Akoto R., Alm L., Drenck T.C., Frings J., Krause M., Frosch K.H. (2020). Slope-Correction Osteotomy with Lateral Extra-articular Tenodesis and Revision Anterior Cruciate Ligament Reconstruction Is Highly Effective in Treating High-Grade Anterior Knee Laxity. Am. J. Sports Med..

[31] Dickschas J., Strobel M.J., Weiler A., Lobenhoffer P., Simon M. (2020). Tibial Slope Correction as an Infratuberosity Closing-Wedge Extension Osteotomy in ACL-Deficient Knees. Z. Orthop. Unf..

[32] Demey G., Mesnard G., Giovannetti de Sanctis E., Dejour D. (2024). A Supratuberosity Anterior Closing-Wedge Proximal Tibial Osteotomy Increases Patellar Height: A Simulated Time Zero Uniplanar Radiographic Study. Arthroscopy.

[33] Luceri F., Basilico M., Batailler C., Randelli P.S., Peretti G.M., Servien E., Lustig S. (2020). Effects of sagittal tibial osteotomy on frontal alignment of the knee and patellar height. Int. Orthop..

[34] Tollefson L.V., Kennedy N.I., Banovetz M.T., Homan M.D., Engebretsen L., Moatshe G., Wulf C.A., Larson C.M., LaPrade R.F. (2024). Supratubercle Anterior Closing Wedge Osteotomy: No Changes in Patellar Height and Significant Decreases in Anterior Tibial Translation at 6 Months Postoperatively. Am J Sports Med.

[35] Christel P., Djian P. (2002). Anterio-lateral extra-articular tenodesis of the knee using a short strip of fascia lata. Rev. Chir. Orthop. Reparatrice Appar. Mot..

[36] Sonnery-Cottet B., Hopper G.P., Gousopoulos L., Pioger C., Vieira T.D., Thaunat M., Fayard J.M., Freychet B., Cavaignac E., Saithna A. (2024). Incidence of and Risk Factors for Arthrogenic Muscle Inhibition in Acute Anterior Cruciate Ligament Injuries: A Cross-Sectional Study and Analysis of Associated Factors From the SANTI Study Group. Am. J. Sports Med..

[37] Sonnery-Cottet B., Ripoll T., Cavaignac E. (2024). Prevention of knee stiffness following ligament reconstruction: Understanding the role of Arthrogenic Muscle Inhibition (AMI). Orthop. Traumatol. Surg. Res..

[38] Ho J.P.Y., Merican A.M., Hashim M.S., Abbas A.A., Chan C.K., Mohamad J.A. (2017). Three-Dimensional Computed Tomography Analysis of the Posterior Tibial Slope in 100 Knees. J. Arthroplast..

[39] Naendrup J.H., Drouven S.F., Shaikh H.S., Jaecker V., Offerhaus C., Shafizadeh S.T., Pfeiffer T.R. (2020). High variability of tibial slope measurement methods in daily clinical practice: Comparisons between measurements on lateral radiograph, magnetic resonance imaging, and computed tomography. Knee.

[40] Lipps D.B., Wilson A.M., Ashton-Miller J.A., Wojtys E.M. (2012). Evaluation of different methods for measuring lateral tibial slope using magnetic resonance imaging. Am. J. Sports Med..

[41] Ihle C., Ahrend M., Grunwald L., Ateschrang A., Stockle U., Schroter S. (2017). No change in patellar height following open wedge high tibial osteotomy using a novel femur-referenced measurement method. Knee.

[42] Caton J., Deschamps G., Chambat P., Lerat J.L., Dejour H. (1982). Patella infera. Apropos of 128 cases. Rev. Chir. Orthop. Reparatrice Appar. Mot..

[43] Seil R., Muller B., Georg T., Kohn D., Rupp S. (2000). Reliability and interobserver variability in radiological patellar height ratios. Knee Surg. Sports Traumatol. Arthrosc..

[44] Carissimi M., Sautet P., Charre D., Hanak L., Ollivier M., Micicoi G. (2021). Patellar height is not modified after isolated open-wedge high tibial osteotomy without change in posterior tibial slope. Orthop. Traumatol. Surg. Res..

[45] Rodner C.M., Adams D.J., Diaz-Doran V., Tate J.P., Santangelo S.A., Mazzocca A.D., Arciero R.A. (2006). Medial opening wedge tibial osteotomy and the sagittal plane: The effect of increasing tibial slope on tibiofemoral contact pressure. Am. J. Sports Med..

[46] Rozinthe A., van Rooij F., Demey G., Saffarini M., Dejour D. (2022). Tibial slope correction combined with second revision ACLR grants good clinical outcomes and prevents graft rupture at 7-15-year follow-up. Knee Surg. Sports Traumatol. Arthrosc..

[47] Chen J.L., Allen C.R., Stephens T.E., Haas A.K., Huston L.J., Wright R.W., Feeley B.T., Multicenter A.C.L.R.S.G. (2013). Differences in mechanisms of failure, intraoperative findings, and surgical characteristics between single- and multiple-revision ACL reconstructions: A MARS cohort study. Am. J. Sports Med..

[48] Cho J.H., Nam H.S., Ho J.P.Y., Tu N.T., Lee Y.S. (2024). Retro-tubercular Biplanar Medial Opening-Wedge High Tibial Osteotomy Results in Superior Patellofemoral Alignment Versus Supra-tubercular Osteotomy. Arthroscopy.

[49] Mabrouk A., An J.S., Fernandes L.R., Kley K., Jacquet C., Ollivier M. (2023). Maintaining Posterior Tibial Slope and Patellar Height During Medial Opening Wedge High Tibial Osteotomy. Orthop. J. Sports Med..

[50] Ferner F., Lutter C., Dickschas J., Strecker W. (2019). Medial open wedge vs. lateral closed wedge high tibial osteotomy—Indications based on the findings of patellar height, leg length, torsional correction and clinical outcome in one hundred cases. Int. Orthop..

[51] Bin S.I., Kim H.J., Ahn H.S., Rim D.S., Lee D.H. (2016). Changes in Patellar Height After Opening Wedge and Closing Wedge High Tibial Osteotomy: A Meta-analysis. Arthroscopy.

[52] Cance N., Dan M.J., Pineda T., Demey G., DeJour D.H. (2024). Radiographic Investigation of Coronal Plane and Patellar Height and Changes following Tibial Deflection Osteotomy for Correction of Tibial Slope in Combination with ACL Reconstruction. Am. J. Sports Med..

[53] Luceri F., Basilico M., Batailler C., Randelli P.S., Lustig S., Servien E. (2022). The Dynamic Effect of Anterior Cruciate Ligament Deficiency on Patellar Height. Indian J. Orthop..

[54] Trojani C., Micicoi G., Boileau P. (2021). High tibial flexion osteotomy for symptomatic ligamentous genu recurvatum. Orthop. Traumatol. Surg. Res..

[55] Moroni A., Pezzuto V., Pompili M., Zinghi G. (1992). Proximal osteotomy of the tibia for the treatment of genu recurvatum in adults. J. Bone Jt. Surg..

[56] Dierick F., Schreiber C., Lavallee P., Buisseret F. (2021). Asymptomatic Genu Recurvatum reshapes lower limb sagittal joint and elevation angles during gait at different speeds. Knee.

[57] Group M., Cooper D.E., Dunn W.R., Huston L.J., Haas A.K., Spindler K.P., Allen C.R., Anderson A.F., DeBerardino T.M., Lantz B.B.A. (2018). Physiologic Preoperative Knee Hyperextension Is a Predictor of Failure in an Anterior Cruciate Ligament Revision Cohort: A Report From the MARS Group. Am. J. Sports Med..

[58] Guimaraes T.M., Giglio P.N., Sobrado M.F., Bonadio M.B., Gobbi R.G., Pecora J.R., Helito C.P. (2021). Knee Hyperextension Greater Than 5 degrees Is a Risk Factor for Failure in ACL Reconstruction Using Hamstring Graft. Orthop. J. Sports Med..

